# Fusing attention mechanism with Mask R-CNN for instance segmentation of grape cluster in the field

**DOI:** 10.3389/fpls.2022.934450

**Published:** 2022-07-22

**Authors:** Lei Shen, Jinya Su, Rong Huang, Wumeng Quan, Yuyang Song, Yulin Fang, Baofeng Su

**Affiliations:** ^1^College of Mechanical and Electronic Engineering, Northwest A&F University, Yangling, China; ^2^Key Laboratory of Agricultural Internet of Things, Ministry of Agriculture and Rural Affairs, Yangling, China; ^3^Shaanxi Key Laboratory of Agricultural Information Perception and Intelligent Services, Yangling, China; ^4^Department of Computing Science, University of Aberdeen, Aberdeen, United Kingdom; ^5^College of Enology, Northwest A&F University, Yangling, China

**Keywords:** grape, instance segmentation, Mask R-CNN, attention mechanism, Dense Upsampling Convolution

## Abstract

Accurately detecting and segmenting grape cluster in the field is fundamental for precision viticulture. In this paper, a new backbone network, ResNet50-FPN-ED, was proposed to improve Mask R-CNN instance segmentation so that the detection and segmentation performance can be improved under complex environments, cluster shape variations, leaf shading, trunk occlusion, and grapes overlapping. An Efficient Channel Attention (ECA) mechanism was first introduced in the backbone network to correct the extracted features for better grape cluster detection. To obtain detailed feature map information, Dense Upsampling Convolution (DUC) was used in feature pyramid fusion to improve model segmentation accuracy. Moreover, model generalization performance was also improved by training the model on two different datasets. The developed algorithm was validated on a large dataset with 682 annotated images, where the experimental results indicate that the model achieves an Average Precision (AP) of 60.1% on object detection and 59.5% on instance segmentation. Particularly, on object detection task, the AP improved by 1.4% and 1.8% over the original Mask R-CNN (ResNet50-FPN) and Faster R-CNN (ResNet50-FPN). For the instance segmentation, the AP improved by 1.6% and 2.2% over the original Mask R-CNN and SOLOv2. When tested on different datasets, the improved model had high detection and segmentation accuracy and inter-varietal generalization performance in complex growth environments, which is able to provide technical support for intelligent vineyard management.

## Introduction

There is an urgent need to develop new technologies and methods for Precision Viticulture (PV) to ensure a greater efficiency, quality and sustainability of agricultural activities (Santesteban, 2019; Gené-Mola et al., 2020). However, agricultural automation is generally more difficult than industrial automation because of the uncertainties of field conditions, plant structures, and the outdoor environments, which necessitates systems capable of monitoring plant and fruit structure at a fine-grained level (Kirkpatrick, 2019). Proper detection and localization of such structures are critical components of agricultural monitoring, robotics, and autonomous systems (Duckett et al., 2018). For a variety of applications, an accurate fruit detection and localization is required, particularly, for fruit counting and yield estimation (Bargoti and Underwood, 2017). Upon fruits being accurately detected and localized, precision agricultural applications can be conducted for inter and intra-field variability management. Fruit detection can also serve as a precursor for disease and nutrient deficiency monitoring, as well as a critical component of actuation (Barbedo, 2019). For example, automated spraying and harvesting could be developed, which is drawing ever-increasing attention given the shrinking agricultural labor force (Shamshiri et al., 2018). In addition, as many agronomically relevant traits are highly heterogeneous with respect to environmental conditions, fruit detection can also be used for field phenotyping to improve plant research and breeding operations (Milella et al., 2019).

Early research on fruit detection relied on classical feature engineering methods, which included human-designed descriptors based on color, geometric and textural features (Gongal et al., 2015). Based on such features, machine learning techniques such as Bayesian classifiers, support vector machines (SVM), and clustering were applied to perform fruit detection and classification (Wu et al., 2012; Kurtulmuş and Kavdir, 2014). Although these schemes can achieve a high computing speed, they suffer from a limited accuracy under challenging conditions such as crop variability, multi-crop detection, lighting changes, and occlusion issues, among others (Xu et al., 2013).

With the rapid development of deep learning methods, many high-performance computer vision algorithms based on deep neural networks have also been successfully applied to fruit detection, with a higher detection speed and accuracy (LeCun et al., 2015; Yan et al., 2021). For example, Wang and He (2021) developed a YOLOV5s model with channel pruning based on a deep learning approach for a fast and accurate detection of growing apple fruit in their natural environment prior to thinning. Parvathi and Selvi (2021) proposed Faster-RCNN with ResNet-50 algorithm which can achieve a precision of 89.4% for the detection of coconut maturity. Previous detection techniques generally relied on rectangular bounding boxes to identify individual items. For fruits with a regular shape such as oranges and apples, this approach if adequately fitted to the fruit boundaries, could provide estimates of fruit shape and space occupancy (circular shape). However, for grape clusters, rectangular boxes would not properly adjust to the berries.

A step further beyond object detection is instance segmentation (Lin et al., 2014), which can correctly identify berry pixels in the detection box, allowing for a finer fruit characterization. Additionally, occlusions caused by leaves, branches, trunks, and even other clusters can also be addressed properly by instance segmentation, which is very useful for robotic manipulation and other automation tasks (Santos et al., 2020). Pérez-Borrero et al. (2020) proposed a deep learning-based approach for strawberry instance segmentation by designing a new architecture based on Mask R-CNN backbone and Mask networks. Based on the 200 test images, the results maintained competitive results to the original Mask R-CNN in term of mean AP (43.85% vs. 45.36%). However, the dataset in the study was single and the context was relatively simple and therefore not representative. As a result, its generalization performance may not be good enough for practical applications under variable natural conditions. Despite the use of various convolutional neural network (CNN) techniques for fruit recognition, the problem of detecting and segmenting wine grapes clusters from field images is still a very complex challenge due to a variety of relevant factors such as environment lighting, complex backgrounds, large shape variations between grapes clusters and occlusion.

In this study, an improved Mask R-CNN model was proposed to ensure the accuracy of grape detection and segmentation in field environments. The main contributions are listed as below:

1. The Efficient Channel Attention (ECA) mechanism was introduced into the backbone network of Mask R-CNN to enhance the feature extraction capability of the network under complex background conditions.
2. Dense Upsampling Convolution (DUC) was used in pyramid feature fusion instead of the traditional nearest neighbor interpolation upsampling method to obtain more image details and improve model accuracy.
3. The improved Mask R-CNN was trained on two datasets with different acquisition criteria to enhance the model generalization performance.
4. The detection and segmentation performance of the improved Mask R-CNN was compared against state-of-the-art (SOTA) models.

## Materials and methods

### Image preparation

#### Image acquisition

This work focuses on wine grapes of the Chardonnay variety in complex field background environments. The collection site was at a wine grape production demonstration site in Yangling, Shaanxi Province, China. Grape images were acquired prior to harvest in the vineyard using a Sony ILCE-5100 l digital camera from Thailand with a spatial resolution of 3,008 × 1,668 pixels, an aperture value of f/3.2 and an exposure time of 1/60 s. The collected images included two main parts, the Grape-A dataset and the Grape-B dataset.

**Figure 1:**
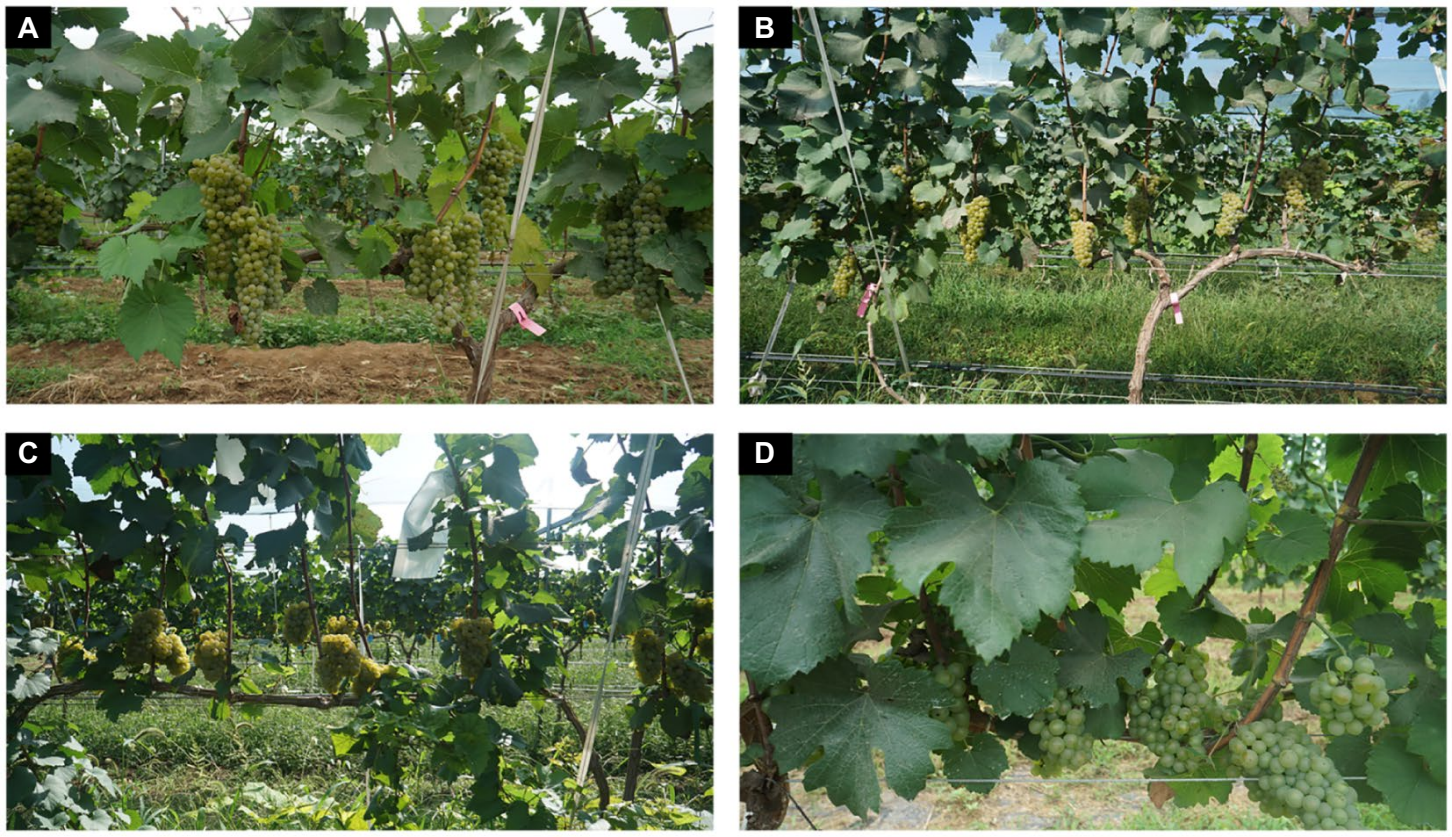
Examples of images acquired under different field sunlight conditions and distances. **(A)** Natural sunlight. **(B)** Smooth light. **(C)** Backlighting. **(D)** Distance changes (2020).

In particular, the Grape-A dataset was collected during July–August 2020 from 9:00 am–12:00 pm each day in a variety of weather conditions such as sunny and cloudy days, and lighting conditions such as down light and back light. The camera lens was randomly placed at a parallel distance of 50–100 cm from the grapes and a total of 218 images were collected. While the Grape-B dataset was collected mainly between July and September 2021. The camera lens was taken at a random distance of 100–150 cm parallel to the grapes, and other acquisition conditions were the same as Grape-A dataset, and a total of 464 images were collected. Figure 1 shows examples of the acquired images under different environmental conditions.

#### Images annotation

**Figure 2:**
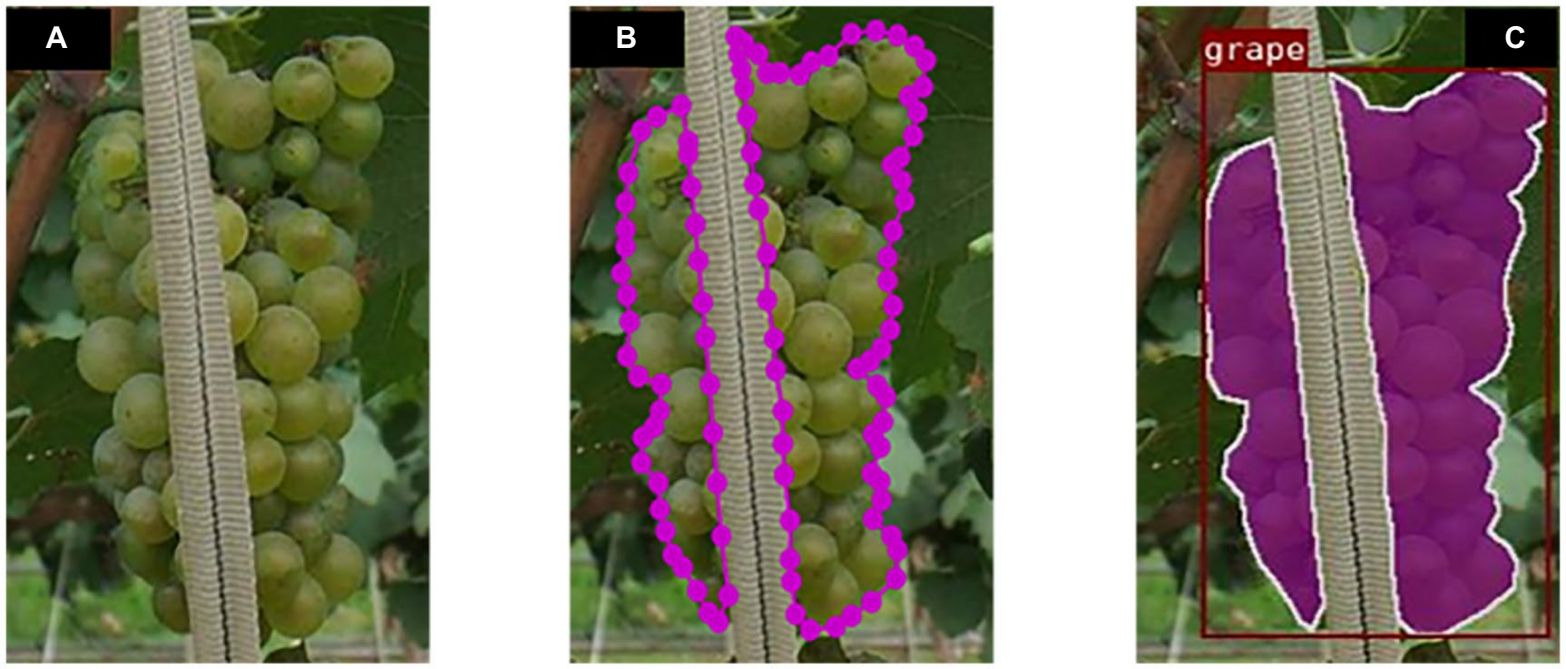
Example annotation of an occluded grape cluster for instance segmentation. **(A)** Divided clusters of the grape. **(B)** Annotation process. **(C)** Instances visualization.

The acquired images were annotated *via* the interactive polygon tool in the LabelMe software (Russell et al., 2008), where the annotation information was saved in JSON files. The tool defines the continuous or discontinuous contours of the grapes by using a sequence of points. The criteria adopted in the labelling process included creating the most accurate possible mask for each cluster of grapes in the image, with the labelled pixels named “grape” consistently and the others treated as background. It covered extreme cases such as clusters that are obscured by trunks, leaves, wires, and ropes, overlapping clusters, and clusters located at the edge of the image or barely perceptible clusters. In cases where occlusion leaves the same target cluster in a truncated or separated state, the difficulty of annotating the same truncated instance can be effectively resolved by setting the same Group id in annotating the image (Figure 2).

**Figure 3:**
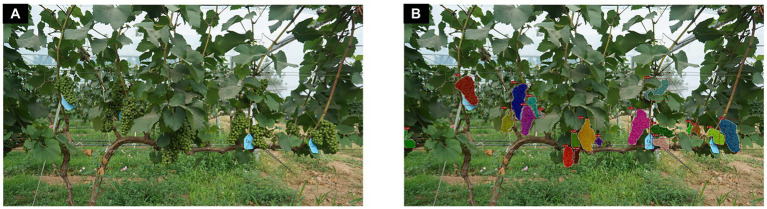
Visualization of the annotation results for an example image. **(A)** Original image. **(B)** Annotated image.

After a precise annotation of each grape cluster contours in each image, the computer program automatically calculates the outer rectangular box based on the cluster polygon contours to save the time required for box annotation in object detection. The visualization of the annotated instance segmentation dataset is illustrated in Figure 3.

### Grape cluster instance segmentation based on improved Mask R-CNN

**Figure 4:**
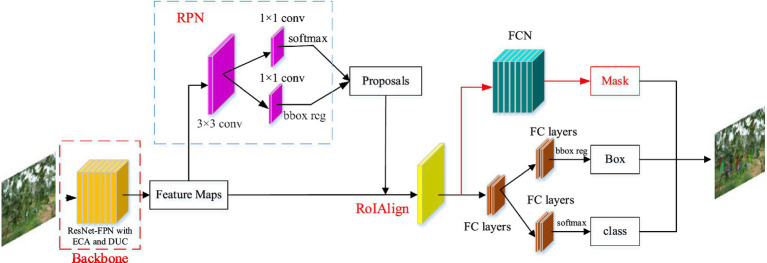
Structure of grape cluster instance segmentation network based on improved Mask R-CNN.

Mask R-CNN (He et al., 2017) is developed on the basis of object detection network Faster R-CNN (Ren et al., 2015), which replaces the RoIPool layer of the original Faster R-CNN with RoIAlign, and uses bilinear interpolation to eliminate the error caused by two quantization operations to solve the problem of region of interest (RoI) mismatch between the feature map and the original image. Additionally, the fully convolutional network (FCN; Long et al., 2015) is added as a semantic segmentation branch of the network. In this study, a grape instance segmentation method based on an improved Mask RCNN was proposed to accurately segment grapes in natural growth environments in the field. A residual feature pyramid structure (ResNet-FPN) fusing Efficient Channel Attention (ECA) and Dense Upsampling Convolution (DUC) were used instead of the original Mask RCNN backbone network to extract grape image features at different scales, and the extracted image features at different scales were used to find the anchor frames of interest in the feature space by the region proposal network (RPN; Li et al., 2018), i.e., rectangular boxes that may contain regions of interest. The network is then split into two branches, classification prediction and mask prediction, where the classification branch is the same as the Faster R-CNN, giving predictions for the RoI and generating category labels and rectangular box coordinates. The mask branch generates a binary mask that depends on the classification results, based on which grape clusters are segmented. Figure 4 shows the structure of the segmenting grape cluster method based on the improved Mask R-CNN network. The details are elaborated in the following subsections.

#### Backbone network

The backbone network is crucial for feature extraction, and sufficient feature information can be extracted from the image to facilitate the subsequent image processing tasks. As the depth of the network increases, the mode performance will gradually degrade. This problem can be effectively solved by introducing a deep residual network (ResNet; He et al., 2016) into the Mask R-CNN backbone.

In this study, two different acquisition criteria were set, and the grape cluster size showed great variability due to the variation of the distance of the camera lens. In the image feature extraction process, the low-level features often contain more detailed information such as texture, color, and contour of the image, but contain a lot of irrelevant information and noise. While the higher-level features often contain high-level semantic information such as category and attributes of the image, but the spatial resolution of the higher-level features is very low, and more detailed information of the image are lost compared with the lower-level features. Therefore, in order to better obtain the grape cluster image feature information, the Feature Pyramid Network (FPN; Kim et al., 2018) is introduced in ResNet, so as to achieve effective integration of low-level features to high-level features at multiple scales, thus making full use of the feature information extracted by convolutional neural networks at different scales.

**Figure 5:**
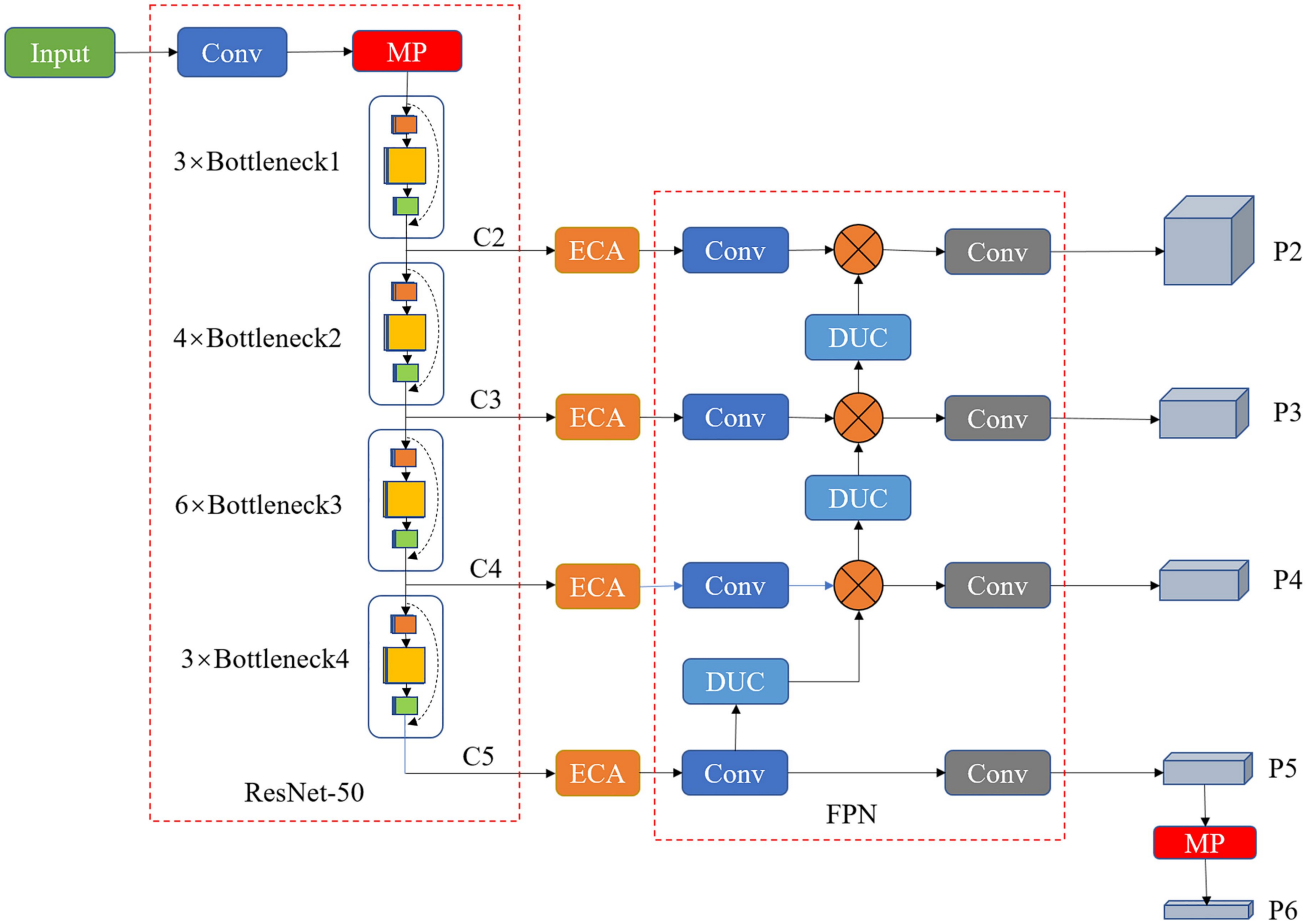
Structure of the improved ResNet50-FPN-ED backbone network.

However, in complex field background environments, the images are susceptible to natural background variations. In particular, grape clusters in natural states have a great close-field nature and overlapping blockage between fruit targets, tree trunk and leaf cover, and light conditions cause some difficulties in the detection and segmentation of grape clusters. The attention-based approach allows the model to focus on and enhance effective feature information while ignoring some useless feature information, thus improving the model’s robustness to field environment variations. In view of the corrective effect of the ECA module on channel features, this study improves the backbone of Mask R-CNN by proposing a new backbone feature extraction network, ResNet50-FPN-ED. The network incorporates an attention mechanism that employs DUC in feature pyramid fusion instead of the traditional nearest neighbor interpolation up sampling method. The ECA module and DUC operations are described as detailed in sections ECA module and Dense Upsampling Convolution, respectively. The structure of the improved backbone network is shown in Figure 5.

In the improved backbone network, the input image is first compressed to 1/2 of the original height (H) and width (W) by a 7 × 7 convolutional kernel in ResNet50, and then the feature maps are compressed to 1/4, 1/8, 1/16, and 1/32 times of the original image size by four different numbers of Bottleneck structures of the residual network to obtain feature maps C2, C3, C4, and C5 with different scales. Besides, these four types of feature maps are input to the channel attention module ECA for feature correction and filtering, and the effective feature information extracted by the network is given a higher weight to complete the calibration of the channel weight information and filter the useless information. The captured grape images have different sizes of grape cluster targets due to the influence of distance variations, and different targets have different features. Therefore, the FPN structure is constructed by connecting the obtained feature maps at different scales horizontally from bottom to top, and feature fusion at different scales is carried out. The fused feature maps at different resolutions are used to detect objects at the corresponding resolution sizes, which ensures that each network layer has the appropriate resolution and strong semantic features. The four feature maps obtained by the ECA module are first uniformly dimensioned down to 256 channels by 1 × 1 convolution, while the output size is maintained. The bottom feature map is then passed through a new up-sampling DUC in a top-down manner to obtain a feature map of the same size, and then the features are fused. This produces a multi-scale feature representation in a single cyclic operation, effectively fusing the information extracted at a deeper level with the feature information extracted by the network at a shallower level. Next, all levels of feature maps are allowed to be fused with features of different resolutions and semantic strengths, resulting in features with both good spatial and strong semantic information. Finally, the fused features of all levels are convolved by a convolution kernel of 3 × 3 size to eliminate the aliasing effect generated by feature fusion. The output is P2, P3, P4 and P5, and P6 is obtained by maximum Pooling of P5 with a stride of 2. These five different scales of feature maps are then used as input to the RPN to find regions of interest. The detailed network structure parameters of the improved backbone are shown in Table 1.

**Table 1:** Parameters of the improved Mask R-CNN backbone: ResNet50-FPN-ED network architecture.

	ResNet-50		ECA		FPN		
Layers		Output size		Conv	Up-sampling	Conv	Output size
Conv1	$\left(\begin{array}{c} 7\times 7,64\\ \mathrm{MP},2 \end{array}\right)$	W/2 × H/2 × 3					
Conv2_x	$\left(\begin{array}{c} 1\times 1,64\\ 3\times 3,64\\ 1\times 1,256 \end{array}\right)\times 3$	C2(W/4 × H/4 × 256)	ECA(256,k_1_)	(1 × 1,256)	ADD	(3 × 3,256)	P2(W/4 × H/4 × 256)
Conv3_x	$\left(\begin{array}{c} 1\times 1,128\\ 3\times 3,128\\ 1\times 1,512 \end{array}\right)\times 4$	C3(W/8 × H/8 × 512)	ECA (512, k_2_)	(1 × 1,256)	DUC(256,1,024)			
					ADD	(3 × 3,256)	P3(W/8 × H/8 × 256)
Conv4_x	$\left(\begin{array}{c} 1\times 1,256\\ 3\times 3,256\\ 1\times 1,1024 \end{array}\right)\times 6$	C4(W/16 × H/16 × 1,024)	ECA(1,024, k_3_)	(1 × 1,256)	DUC(256,1,024)			
					ADD	(3 × 3,256)	P4(W/16 × H/16 × 256)
Conv5_x	$\left(\begin{array}{c} 1\times 1,512\\ 3\times 3,512\\ 1\times 1,1024 \end{array}\right)\times 3$	C5(W/32 × H/32 × 2048)	ECA(2048, k_4_)	(1 × 1,256)	DUC(256,1,024)	(3 × 3,256)	P5(W/32 × H/32 × 256)	
					(3 × 3,256)	(MP,2)	P6(W/64 × H/64 × 256)

k is the convolutional kernel size in the ECA module, (MP,2) are maximum pooling, and stride = 2. ADD is the feature map fusion operation.

#### ECA module

**Figure 6:**
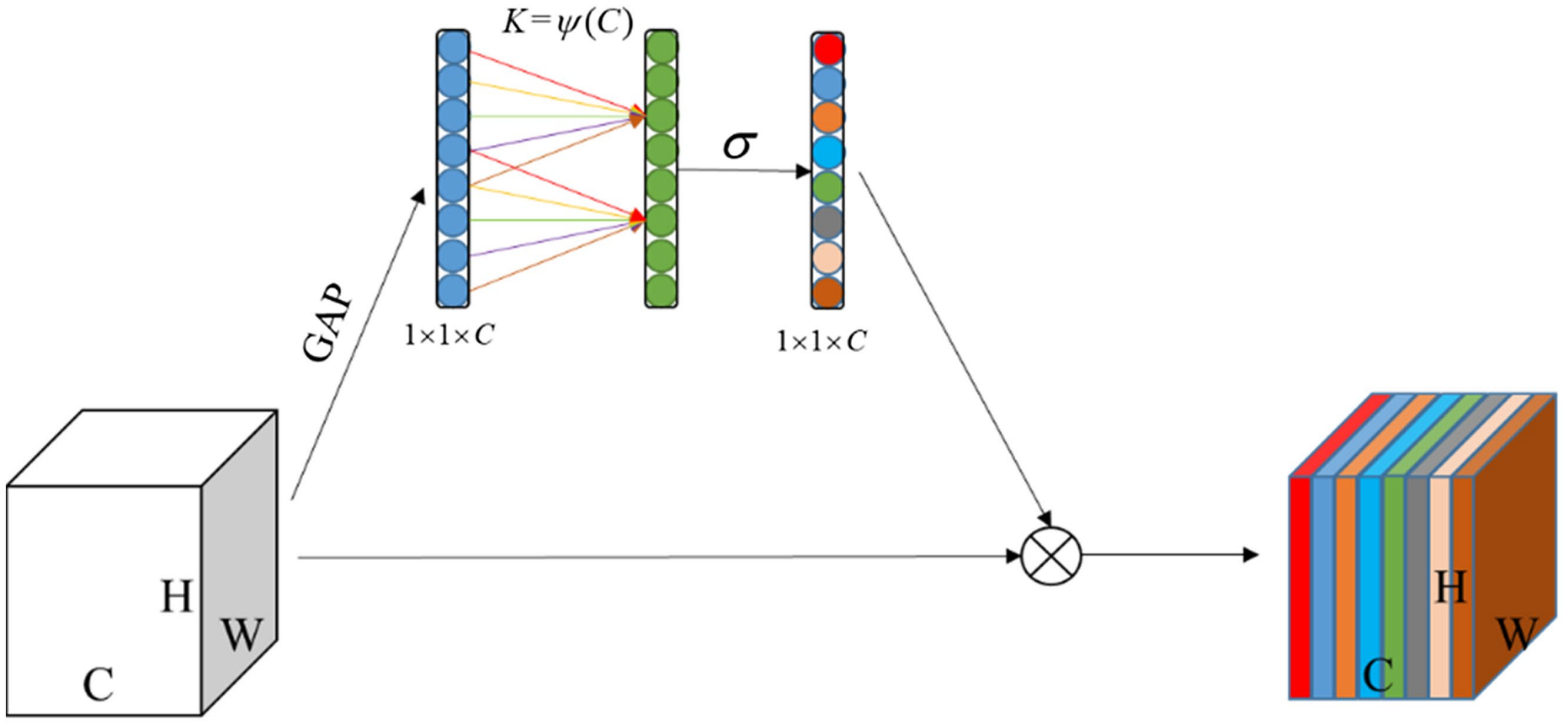
ECA Module.

The SE (Hu et al., 2018) module brings side effects to channel attention prediction by performing a fully-connected operation after dimensionality reduction of the channels, as capturing the dependencies between all channels is inefficient and unnecessary. The ECA (Wang et al., 2020a) module, as a channel attention module, is an enhanced and improved version of the SE module, where its structure is shown in Figure 6. Its main idea is to propose a local cross-channel interaction strategy without dimensionality reduction, which captures local cross-channel interaction information by considering each channel and its k-nearest neighbors after global average pooling (GAP) of channels. The ECA module firstly computes the input feature map of size H × W × C (C is number of feature channels) using GPA to obtain a feature vector of size 1 × 1 × C to have a global receptive field. Secondly, the cross-channel interaction information was captured by 1D convolution with a convolution kernel size of k. The convolution kernel size k was related to the number of input channels and was adaptively chosen by Eq. (1) to determine the coverage of local cross-channel interactions (Wang et al., 2020a). The weights of each feature channel are then generated by a sigmoid activation function calculation. Finally, the output feature channel weight vector is multiplied with the original input feature map to complete the original feature calibration in the channel dimension, so that the extracted features are more directional, and invalid or ineffective feature channels are suppressed, thus improving the extraction of effective features. The ECA module avoids dimensionality reduction operations allowing the model to learn more effective channel attention, and the module has a small number of parameters which is determined only by its convolutional kernel size K (almost negligible).


(1) $$\psi \left(C\right)={\left|\frac{\log_{2}\left(C\right)}{\gamma }+\frac{b}{\gamma }\right|}_{odd}$$

where C is the number of input channelst, ${\left|t\right|}_{odd}$ is an odd number similar to t, γ was set to 2 and b was set to 1.

#### Dense Upsampling Convolution

**Figure 7:**
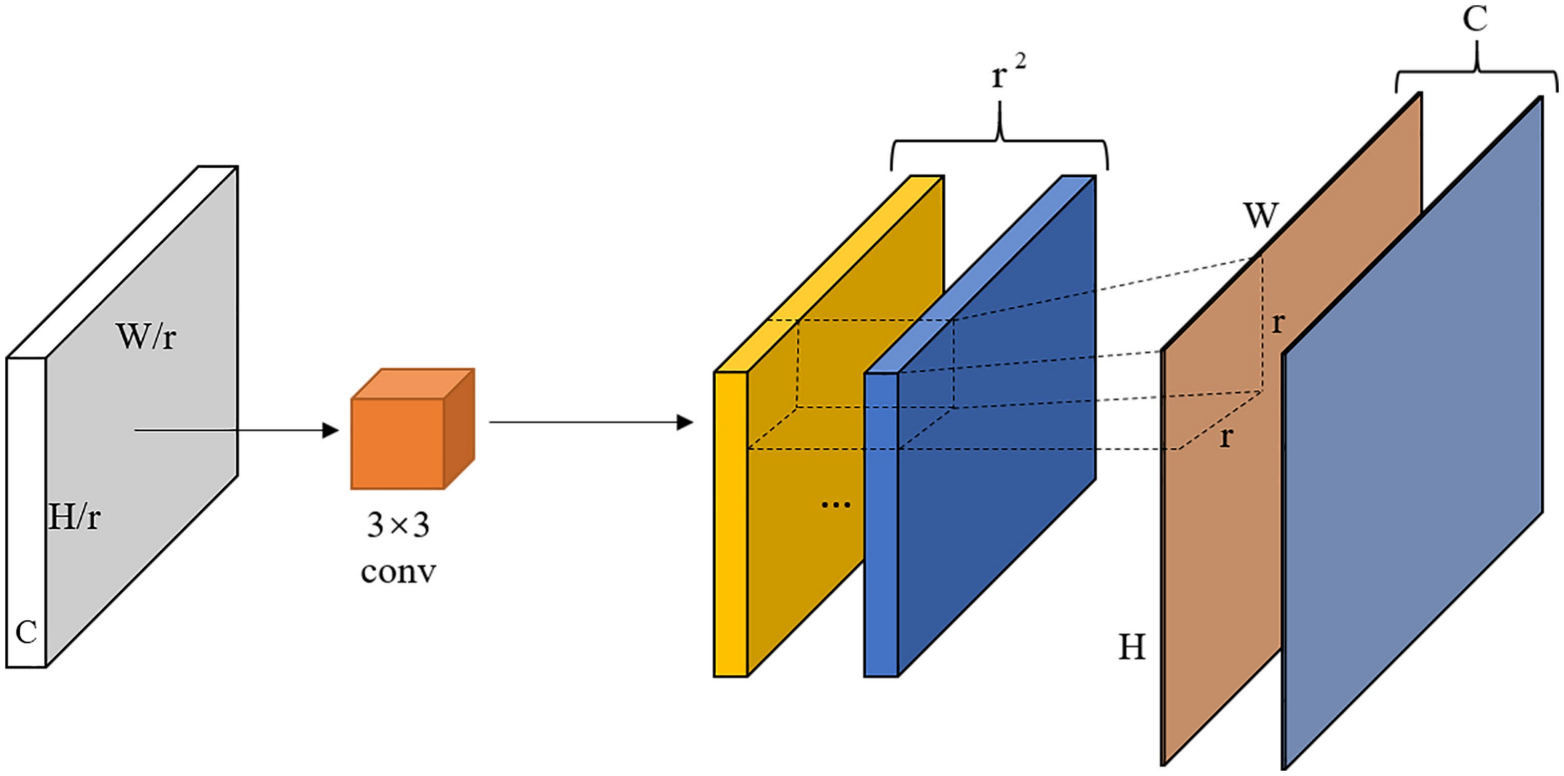
Illustration of DUC operation.

When FPN fuses feature maps from the bottom up, the resolutions of feature maps obtained from different stages and depth networks are different (e.g., the output size of C2, C3, C4, C5), so upsampling operations are required to obtain feature maps of the same size as the previous level and then add them pixel by pixel for feature fusion to build a feature pyramid structure. However, the original Mask R-CNN uses a simple nearest neighbor interpolation approach for upsampling, which results in the loss of detailed information of some features and the interpolation is unlearnable, which has side effect on the model performance. Inspired by super-resolution, this paper uses DUC (Wang et al., 2018) instead of nearest neighbor interpolation upsampling operation, which is able to compensate for the loss in aspect dimension by channel dimension. The feature map is restored to the required resolution by making the model learn a series of upsampling convolution filters. The specific DUC operation flow is shown in Figure 7. The obtained feature map (H/r × W/r × C) is first fed into a set of 3 × 3 convolutions for learning, and the size of the feature map obtained after convolution is H/r × W/r × (C × r^2^), and then reshaped to H × W × C size, where r is the ratio of the up-sampled recovered feature map to the original feature map size.

#### Grape cluster instance segmentation and loss function

Feature maps of five size scales generated by the improved backbone extraction network are sent to the RPN network. The size of grape clusters in different images varies greatly due to different capture distances. Five different scale anchors were designed in generating RoI: 8, 16, 32, 64, and 128, combined with labeling rectangular boxes with three aspect ratios of 0.5, 1, and 2. The final combination of 15 benchmark windows for predicting the regions containing grape clusters in the images makes the output more accurate RoI. The generated RoI and the corresponding feature maps are then sent to RoIAlign for alignment and fixing the anchor size. Finally, the aligned feature maps are passed to t the fully connected layer and fully convolutional layers. The fully connected layer is responsible for performing bounding box regression and classification, while the fully convolutional layer implements grape cluster instance segmentation by deconvolution operations to generate mask.

The loss function is essential for model training, which allows the model’s weights to be continuously optimized by the difference between prediction and ground truth. The loss function of the improved Mask R-CNN is shown in Eq. (2).


(2) $$L=L_{cls}+L_{box}+L_{mask}$$

where $L_{cls}$ is the classification loss, $L_{box}$ is the regression loss of the bounding box, and $L_{mask}$ is the mask loss. In particular, the mask branch generates a mask of size m x m for each RoI and each category, for a total of n categories. Thus, the total output is of size n×m×m. For the predicted binary mask output, a sigmoid function is applied to each pixel point and the resulting result is used as input to the cross-entropy loss function, with the overall loss defined as the average binary cross-loss entropy.

### Model training

The experiments were conducted by using the open source platform detectron2, Pytorch version 1.7.1, with an NVIDIA RTX 3090 graphics card, CUDA version 11.0, and 24 GB of video memory. All training and testing of the models were performed on a Linux (Ubuntu 20.04) operating system.

The datasets (images and annotation results) under two different conditions were divided into a training set and a test set with equal proportions of 8:2, where the detailed division results are shown in Table 2. In order to facilitate subsequent model training and testing, the annotated results of each part of the dataset were converted to the annotation style of the standard COCO dataset (Lin et al., 2014).

**Table 2:** Training set and test set splitting.

	Dataset name	Images	Masked clusters
Train	Grape-A	174	689
	Grape-B	370	4,244
	Total	544	4,933
Test	Grape-A	44	219
	Grape-B	94	1,075
	Total	138	1,294

In order to speed up the model training, weights trained on the COCO dataset were adopted. For the improved Mask R-CNN model, as its network structure has been changed accordingly, the network is initialized by loading partial weights. The hyperparameters for model training were set to 50 for epoch, 2 for batch size, 0.01 for the initial learning rate, and 0.1 times the initial value for decay rate for every 5,000 iterations. To prevent model overfitting, weight decay was set to 10^−4^ and stochastic gradient descent (SGD; Bottou, 2012) was used to update the parameters and optimize the training process.

Data enhancement techniques were used randomly during the training process, meaning that each new batch of images was input to the network for training with random mirroring operations (horizontal and vertical), rotation, cropping and color changes (brightness, contrast and saturation) applied online to the input images, along with a transformation of the coordinate information in the corresponding annotation file. In addition, a random scaling process was set for each batch of images with a minimum edge length from 640 to 800 pixels, in steps of 32, and a maximum edge size of no more than 1,443 pixels.

### Model evaluation metrics

Similarly, COCO competition metrics (Lin et al., 2014) including average precision (AP) and average recall (AR) were used to evaluate the performance of the proposed grape cluster instance segmentation model. The AP summarizes the shape of the precision/recall curve, and is defined as the mean precision at a set of equally spaced recall levels (Everingham et al., 2010). AR is the maximum recall given a fixed number of detections per image, averaged over categories and *intersection over unions* (IoU). The necessary metrics including precision (P) and recall (R) in the calculation of AP and AR are described by Eq. (3).


(3) $$P=\frac{TP}{TP+FP},\;R=\frac{TP}{TP+FN}$$

where TP, FP, and FN means true positive, false positive, and false negative, respectively.

In instance segmentation, a prediction is considered a TP when it IoU is greater than a selected threshold, otherwise it is considered a FP. Some of the metrics used in the COCO competition are shown in Table 3 (Tassis et al., 2021).

**Table 3:** COCO metrics definition.

Metric	Definition
AP	The average of these 10 tests is then used as the final value for each 0.05 change from IoU= 0.5 toIoU= 0.95
$AP_{IoU=0.5}$	Precision is calculated considering IoU > 0.5 for TP
$AP_{IoU=0.75}$	Precision is calculated considering IoU > 0.75 for TP
$AR_{\max=1}$	Recall considering only the detection of an object
$AR_{\max=10}$	Recall considering the detection of up to 10 objects
$AR_{\max=100}$	Recall considering the detection of up to 100 objects

## Results

### Overall test results of the improved Mask R-CNN model

In order to evaluate the performance of the improved Mask R-CNN model for grape recognition under complex background conditions, a test dataset was created with a total of 138 images from both Grape-A and Grape-B datasets acquired in different years. The performance of the ResNet50-FPN, ResNet101-FPN and the Mask R-CNN with ResNet50-FPN-ED (an improved backbone network in this paper) was first evaluated for grape clusters recognition. The results on the test set are shown in Table 4, which show that the proposed improved Mask R-CNN backbone ResNet50-FPN-ED has significant advantages over the original backbone of Mask R-CNN. In particular, the AP reaches 60.10% on object detection task with an improvement of 1.4% over the original ResNet50-FPN; when IoU = 50%, the $AP_{IoU=0.5}$ reaches 85.60% with an improvement of 0.5%; for more stringent metrics, when IoU= 75%, $AP_{IoU=0.75}$ with an improvement of 1.1%; $AR_{\max=100}$ reached 69.50% with an improvement of 2.10% over the original backbone. On the instance segmentation task, AP reached 59.50% with an improvement of 1.6% over the original backbone, $AP_{IoU=0.5}$ reached 87.10% with an improvement of 0.8%, $AP_{IoU=0.75}$ reached 66.90% with an improvement of 2.60%, $AR_{\max=100}$ reached 66.90% with an improvement of 1.80% over the original backbone. Although the average inference speed of the proposed method on the test set is 62.6 ms per frame, which is slightly longer than the inference time of the original Mask R-CNN with ResNet50-FPN (57.3 ms per frame), it effectively improves the detection and segmentation accuracy for grape clusters. In addition, it was found that the trained Mask R-CNN with a deeper backbone ResNet101-FPN did not bring a greater improvement in detection and segmentation accuracy in field grape cluster recognition. The results of using ResNet50-FPN-ED as the Mask R-CNN backbone in this study were significantly better than ResNet101-FPN. Moreover, the deeper ResNet101-FPN network model has a much larger number of parameters, which takes a longer time to train and make inference (with instance segmentation speed being only 70.4 ms per frame), and therefore is not scalable. As the IoU increases, the AP value decreases, which is in line with the actual detection results of the model. It is also noted that the $AR_{\max=1}$ is lower for both tasks due to the fact that all images contain multiple instances of the object of interest, especially for the Grape-B dataset, as the camera was captured at a greater distance in image acquisition and multiple grape instances could be observed within the field of view. For both identical tasks, the same Mask-RCNN network as used in the literature presented good results, e.g., Santos et al. (2020) used the same network for grape detection and instance segmentation in a field setting and obtained $AP_{IoU=0.5}$= 74.3%, further confirming that the improved Mask R-CNN model is more accurate while with a higher reliability.

**Table 4:** Test results for different backbones on Mask R-CNN (the best one is highlighted in bold, the same below).

Backbone	ResNet50-FPN		ResNet50-FPN-ED		ResNet101-FPN	
	Box	Seg	Box	Seg	Box	Seg
AP	58.70	57.90	**60.10**	**59.5**	57.90	57.20
$AP_{IoU=0.5}$	85.10	86.30	**85.60**	**87.10**	85.00	86.70
$AP_{IoU=0.75}$	64.00	64.30	**65.10**	**66.90**	61.70	62.60
$AR_{\max=1}$	9.00	8.80	**9.50**	**9.20**	9.30	9.20
$AR_{\max=10}$	56.00	54.70	**57.10**	**55.70**	55.40	54.30
$AR_{\max=100}$	67.40	65.10	**69.50**	**66.9**	66.90	64.40

Box represents the bounding box result of object detection, Seg represents the segmentation result, same as below.

**Figure 8:**
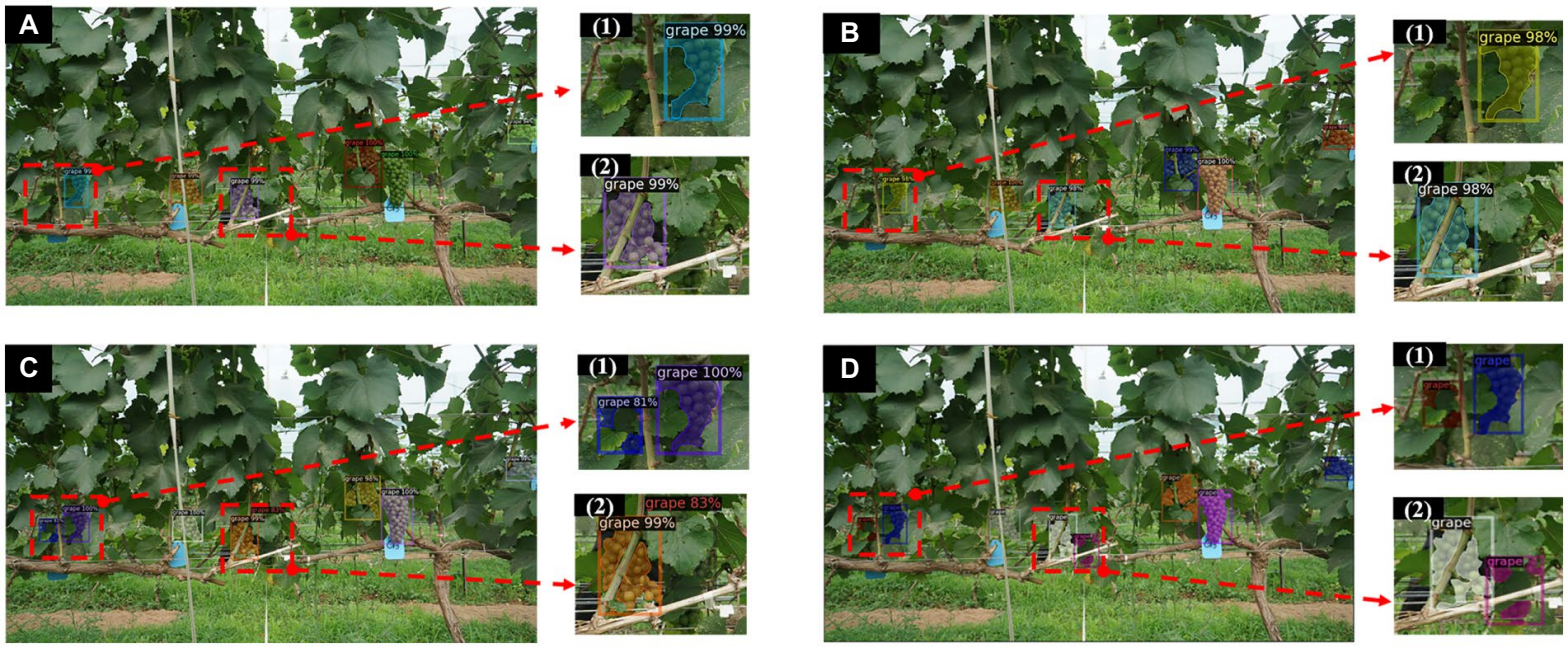
Instance segmentation results by Mask R-CNN with different backbones. **(A)** Instances segmentation results of Mask RCNN with ResNet50-FPN. **(B)** Instances segmentation results of Mask RCNN with ResNet101-FPN. **(C)** Instances segmentation results of improved Mask RCNN with ResNet50-FPN-ED. **(D)** Ground truth results. (1, 2) are partial enlargements of the instance segmentation results of the corresponding images, respectively.

When IoU= 50%, the proposed method achieves an $AP_{IoU=0.5}$of more than 85% for both detection and instance segmentation, but the results may still contain some uncertainty as the annotations are hand-made and not all cluster instances are annotated. This is because the target area where some cluster instances may appear is very small and not easily detected due to severe occlusion and irregular contours of the clusters. Due to the irregular contours of the clusters, some grapes grow too densely for the individual instances to be distinguished from each other when annotating clusters that overlap each other with some subjectivity and uncertainty. Therefore, when comparing the results, the network may have found other instances that were not labelled, or may not be able to indicate the labelled instances. In addition, some grapes grow overly densely resulting in overlap between clusters and shading by trunk leaves, which also affects model recognition. However, the proposed method is able to improve this situation. Figure 8 shows some prediction results of the Mask R-CNN model on the test set with a number of different backbones (including the improved one). The proposed backbone (ResNet50-FPN-ED) is able to detect more instances compared to the other two backbone networks (Figures 8C-1,2).

### Grape segmentation results under different test sets

**Figure 9:**
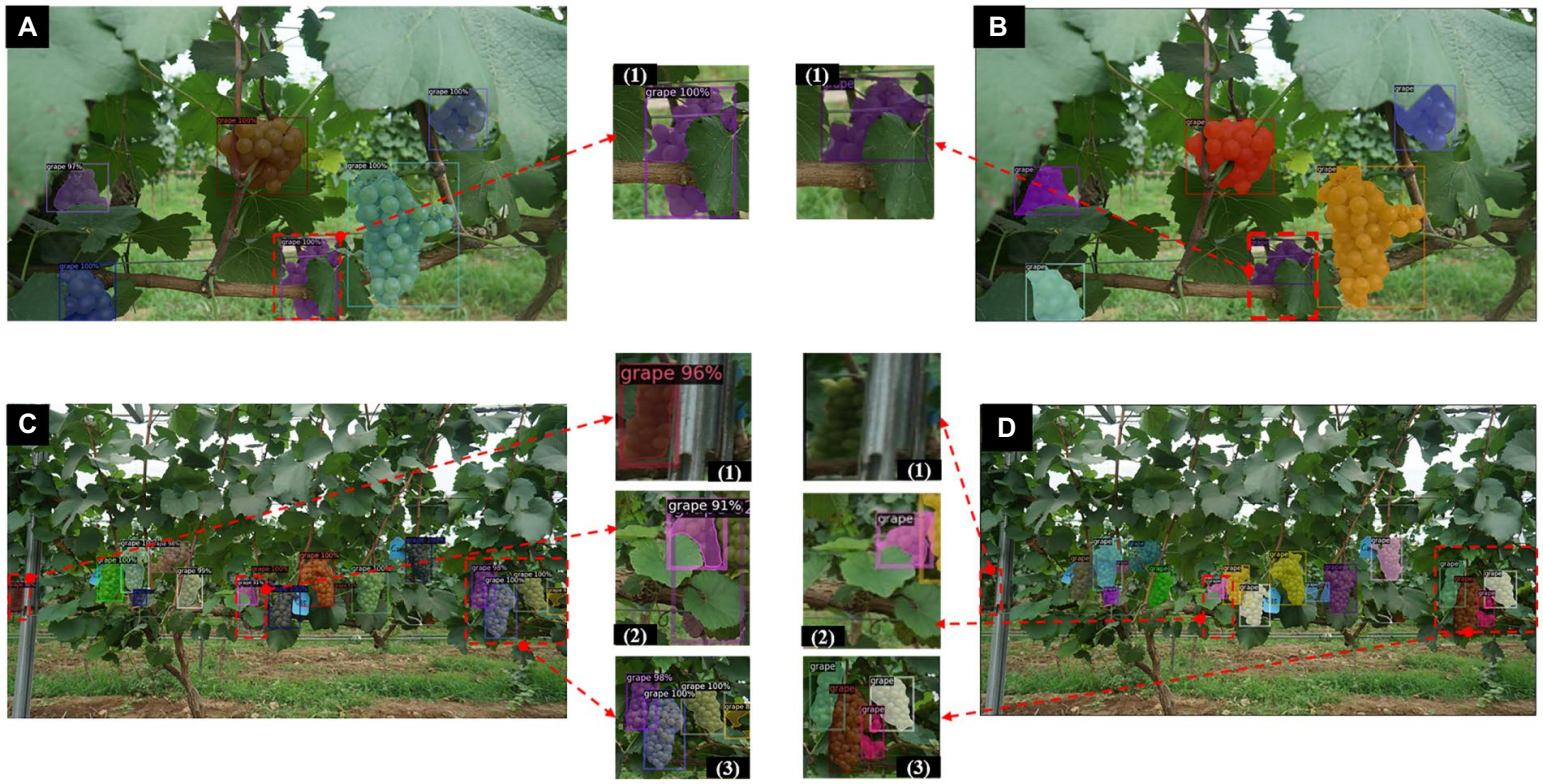
Instance segmentation results of the improved Mask R-CNN on two different types of test datasets. **(A)** Several image segmentation results of improved Mask R-CNN in Grape-A dataset. **(B)** Ground truth for several images in the Grape-A dataset. **(C)** Several image segmentation results of improved Mask R-CNN in Grape-B dataset. **(D)** Ground truth for several images in the Grape-B dataset. (1-3) are partial enlargements of the instance segmentation results of the corresponding images, respectively.

To further explain the performance of the proposed model under two different datasets Grape-A and Grape-B, the test sets of these two datasets were tested separately (i.e., the same training dataset with different test datasets). The example instance segmentation results of the improved Mask R-CNN for the two different types of datasets are shown in Figure 9. Figures 9A-1,C-1–3 show some of the instance regions found by the proposed model that are not fully annotated. This is due to the fact that the dataset was manually annotated and some instances are occluded and at the edges of the image which are difficult to perceive even for the human eye (Figures 9B-1,D-1,3). It can be seen that the number of observable instances on the Grape-A dataset is less than on Grape-B due to the capture distance (Figures 9A,C). Moreover, due to the denser field of view, the cluster shapes vary more significantly between instances in the Grape-B dataset, thus with more areas of cluster overlap. Figures 9C-2 shows that due to the occlusion of the leaves and trunk, the model generates a duplicate segmentation, mistaking two different instances for the same one, and surprisingly also found unannotated instances at the same time. However, the overall segmentation results are promising. It can effectively avoid the missed detection due to occlusion.

The performance of the backbone ResNet50-FPN-ED in the improved Mask R-CNN was compared against the original backbone ResNet50-FPN on the two different datasets. The results on the test set of Grape-A dataset and Grape-B dataset are shown in Tables 5, 6, respectively. It is clearly observed from both tables that the proposed improved Mask R-CNN approach has significantly improved AP values for both detection and instance segmentation tasks over the original Mask R-CNN under two different types of datasets. This further confirms that the proposed method has better detection and segmentation results in dealing with datasets with different capture distances. It is worth noting that, as shown in the table, the proposed method has the most significant improvement in the detection and segmentation tasks on the Grape-A dataset. Compared with the original model, AP, $AP_{IoU=0.5}$, $AP_{IoU=0.75}$, and $AR_{\max=100}$ have improved detection tasks by 2.6%, 0.9, 3.1%, and 2.9%, respectively. The performance for instance segmentation tasks are increased by 2.2%, 1.5%, 0.5%, and 2.3%, respectively. As aforementioned, this is related to the collection standards of the two datasets. Due to the observation distance and camera field of view, the target size of the overall grape cluster instance of Grape-A dataset is larger than that of Grape-B dataset. The introduced attention mechanism ECA module is more sensitive to larger target sizes than small targets, and the improved detection effect is also more significant.

**Table 5:** Performance comparison between the improved Mask R-CNN backbone and the original one on the Grape-A test set.

Backbone	ResNet50-FPN		ResNet50-FPN-ED	
	Box	Seg	Box	Seg
AP	59.90	61.00	**62.50**	**63.20**
$AP_{IoU=0.5}$	81.90	84.70	**82.80**	**86.20**
$AP_{IoU=0.75}$	63.50	71.20	**66.60**	**71.70**
$AR_{\max=1}$	17.40	17.00	**17.80**	**17.30**
$AR_{\max=10}$	64.50	64.20	**67.10**	**66.90**
$AR_{\max=100}$	68.10	68.30	**71.00**	**70.60**

The best one is highlighted in bold.

**Table 6:** Performance comparison between the improved Mask R-CNN backbone and the original one on the Grape-B test set.

Backbone	ResNet50-FPN		ResNet50-FPN-ED	
	Box	Seg	Box	Seg
AP	58.80	57.40	**59.70**	**58.80**
$AP_{IoU=0.5}$	85.50	87.00	**86.50**	**87.20**
$AP_{IoU=0.75}$	64.60	62.90	**64.80**	**66.10**
$AR_{\max=1}$	7.30	7.10	**7.70**	**7.50**
$AR_{\max=10}$	54.20	52.60	**54.90**	**53.40**
$AR_{\max=100}$	67.20	64.40	**69.10**	**66.10**

The best one is highlighted in bold.

### Comparison of the improved Mask R-CNN model with SOTA models

**Figure 10:**
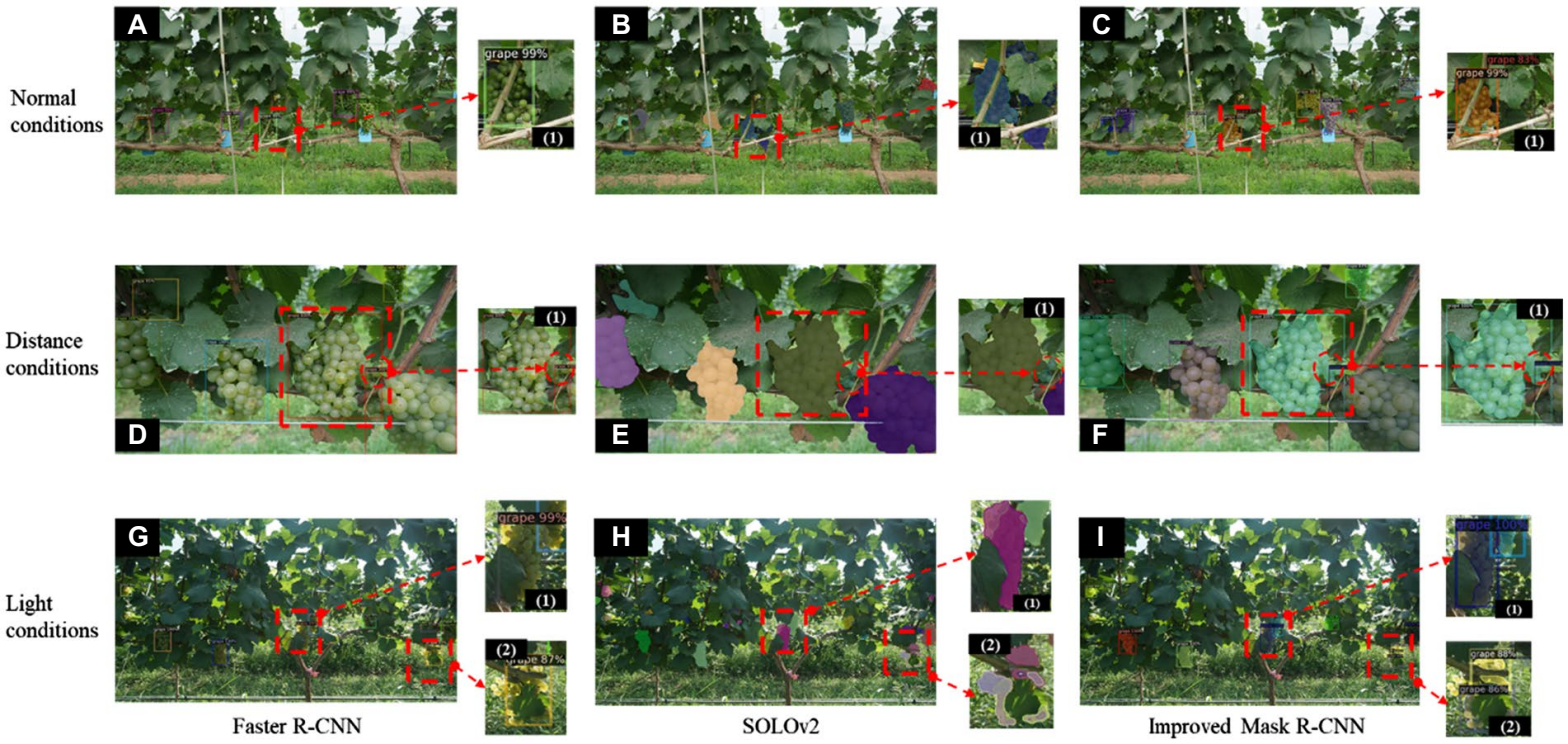
Comparative results of the improved Mask R-CNN model with Faster R-CNN and SOLOv2 under different conditions. **(A–C)** are the prediction results of Faster R-CNN, SOLOv2, and improved Mask R-CNN under normal conditions, respectively. **(D–F)** are the prediction results of Faster R-CNN, SOLOv2, and improved Mask R-CNN under distance conditions, respectively. **(G–I)** are the prediction results of Faster R-CNN, SOLOv2, and improved Mask R-CNN under light conditions, respectively. (1,2) are partial enlargements of the instance segmentation results of the corresponding images, respectively.

To further verify the improved Mask R-CNN model, two SOTA models were compared. The instance segmentation network SOLOv2 (Wang et al., 2020b) and the object detection network Faster R-CNN (ResNet50-FPN) were also trained under the same conditions and compared on the 138 test images, where the results of the post-test comparisons are shown in Table 7. In addition, the model prediction results under different natural conditions were shown in Figure 10. The Faster R-CNN only performs object detection, and the results show that although the detection speed is 50.24 ms per frame, slightly faster than the improved Mask R-CNN model, it does not perform semantic segmentation to classify the berry pixels in detail. Because mask prediction has a time cost. Under the conditions of close camera lens distance, the two object box detections achieve almost the same results (Figures 10D,F), however, the improved Mask R-CNN has an advantage in the case of light variation and more severe occlusion (Figures 10A,C,G-2,I-2). The improved Mask R-CNN has improved AP by 1.8%, $AP_{IoU=0.75}$ by 1.4%, and $AR_{\max=100}$ by 2.3% compared to the Faster R-CNN in object detection task. Although there is no significant difference in $AP_{IoU=0.5}$, the overall detection accuracy of the improved Mask R-CNN is better. In particular, SOLOv2 seems to perform better than Faster R-CNN for instance prediction under normal conditions (Figures 10A,B) but Figures 10H-1,2 shows more false segmentation in bright light. This may be related to the structure of the SOLOv2 network, which transforms the segmentation problem into a location classification problem and deals directly with instance segmentation without relying on box detection. SOLOv2 network is not conducive to the segmentation of overlapping targets (Figures 10E-1) and has a detection speed of only 54.90 ms per frame, which is slower than the proposed model. For the instance segmentation task, the proposed method improves AP by 2.2%, $AP_{IoU=0.5}$ by 3.5%, $AP_{IoU=0.75}$ by 2.9%, and $AR_{\max=100}$ by 2.5% over the SOLOv2. It can be seen that the proposed improved Mask R-CNN model has a higher accuracy and stability than SOLOv2 and Faster R-CNN for grape cluster detection and segmentation under complex background conditions, which further demonstrates the advantages of the proposed method.

**Table 7:** Comparative test results of the improved Mask R-CNN model against Faster R-CNN and SOLOv2.

Model	Faster R-CNN	SOLOv2	Improved Mask R-CNN	
	Box	Seg	Box	Seg
AP	58.30	57.30	**60.10**	**59.5**
$AP_{IoU=0.5}$	**85.80**	83.60	85.60	**87.10**
$AP_{IoU=0.75}$	63.70	64.00	**65.10**	**66.90**
$AR_{\max=1}$	9.20	**9.30**	**9.50**	9.20
$AR_{\max=10}$	55.50	54.90	**57.10**	**55.70**
$AR_{\max=100}$	67.20	64.40	**69.50**	**66.9**

The best one is highlighted in bold.

## Discussion

### Effect of different natural environmental conditions on grape segmentation

**Figure 11:**
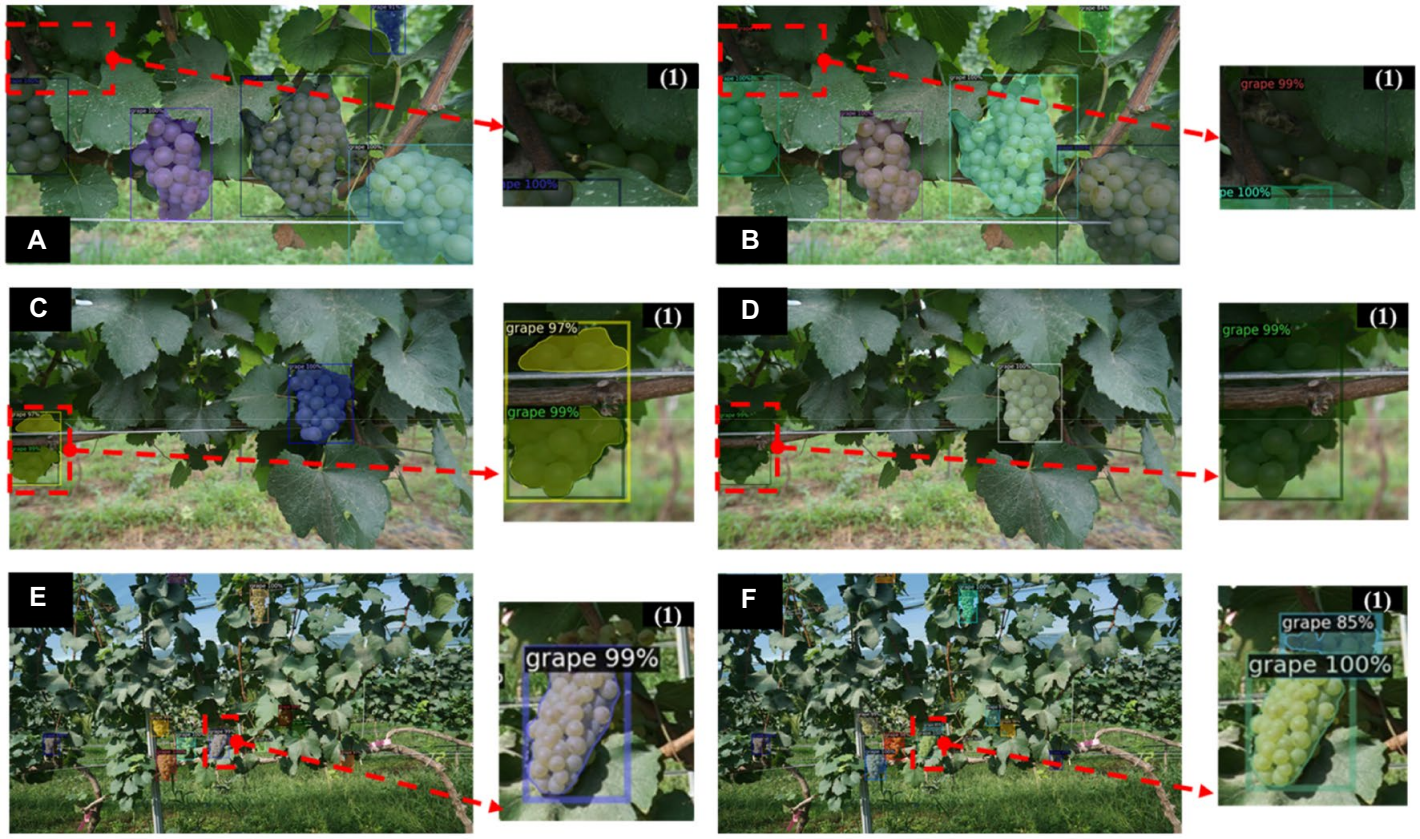
Effects of different natural environmental conditions on the performance of the improved Mask R-CNN. **(A,C,E)** are the predictions of the original Mask R-CNN under low light, occlusion and overlapping grape clusters in smooth light, respectively. **(B,D,F)** are the predictions of the improved Mask R-CNN under low light, occlusion and overlapping grape clusters in smooth light, respectively. (1) is partial enlargements of the instance segmentation results of the corresponding images.

Based on the image detection and instance segmentation of grape clusters, the model detection results show some variability due to the huge variation of light conditions in the field background conditions and the variation of camera lens distance during data collection. To verify the effectiveness of the improved Mask R-CNN under varying light conditions and camera lens variation in field conditions, the original Mask R-CNN was chosen to compare the instance segmentation performance. The results are shown in Figure 11. It can be seen that the original Mask R-CNN model results in missed detection due to darker regions caused by lighting and camera viewpoint changes, and overlapping occlusion between grape clusters (Figures 11A,E). In addition, the same grape instance is divided into two parts caused by the occlusion of the wire and the tree trunk, which leads the model to incorrectly detect one instance as two (Figure 11C). However, the proposed improved Mask R-CNN method can recover more instances than the original model under variable complex conditions (Figures 11B,F), and Figures 11D-1 also shows that the model is potentially corrective to the extracted features, indicating that the method introducing the channel attention mechanism can extract more effective feature information and suppress useless feature information than the original backbone, thus improving the model detection and segmentation accuracy. This result is consistent with that of Jiang et al. (2022) who improved the detection accuracy of young apple in low-quality images by adding the Non-local attention module (NLAM) and Convolutional block attention model (CBAM) to the baseline of the YOLOv4 model. The experimental results also show that the attention mechanism can better improve the detection accuracy of images with highlights/shadows, and severe occlusions. Previous studies (Li et al., 2021; Wang and He, 2022) have similarly shown this result. In particular, in this study, the DUC operation employed for feature fusion can recover more image detail information from the previous level in the feature fusion stage, which is very effective for semantic segmentation tasks.

### Analysis of grape segmentation results under different training datasets

To assess the effect of models trained on different types of datasets, the improved Mask R-CNN was trained and tested individually on Grape-A and Grape-B datasets, and compared with the original Mask R-CNN, where the comparative results for Grape-A dataset and Grape-B dataset are shown in Tables 8, 9, respectively. It is shown that the proposed method consistently outperforms the original method. Notably, comparing the results of the models built on the overall dataset in section “Grape segmentation results under different test sets” and the two distinct types of test sets, the test results on dataset Grape-A alone were superior to the test results on a combination of the two datasets. However, the test results of the models trained on dataset Grape-B alone were almost remained consistent, which might be related to the structure of the training dataset. The great variability and specificity of the features between the samples inevitably introduces some adversarial and redundant features into the model learning process, leading to a lower performance of the combined training model than if it had been trained on a single dataset. Also, the model constructed from a single dataset suffered from poor generalization performance to more heterogeneous datasets. Although the number of grape cluster instances in Grape-A dataset was small, it was sufficient for the model to learn enough features to identify grape clusters under the same environmental conditions.

**Table 8:** Comparison of test results with different backbones based on dataset Grape-A.

Backbone	ResNet50-FPN		ResNet50-FPN-ED	
	Box	Seg	Box	Seg
AP	63.70	63.00	64.80	63.80
$AP_{IoU=0.5}$	83.60	86.20	84.70	85.70
$AP_{IoU=0.75}$	70.90	73.00	72.20	72.00
$AR_{\max=1}$	17.00	16.70	17.90	17.20
$AR_{\max=10}$	68.40	66.40	67.70	66.10
$AR_{\max=100}$	73.00	70.70	73.00	71.00

**Table 9:** Comparison of test results with different backbones based on dataset Grape-B.

Backbone	ResNet50-FPN		ResNet50-FPN-ED	
	Box	Seg	Box	Seg
AP	58.30	57.20	59.40	58.40
$AP_{IoU=0.5}$	86.10	87.00	87.20	87.80
$AP_{IoU=0.75}$	62.70	63.30	64.70	64.90
$AR_{\max=1}$	7.50	7.30	7.60	7.30
$AR_{\max=10}$	54.10	52.50	54.30	53.40
$AR_{\max=100}$	66.60	63.80	68.80	66.60

### Effect of different grape varieties on grape segmentation

**Figure 12:**
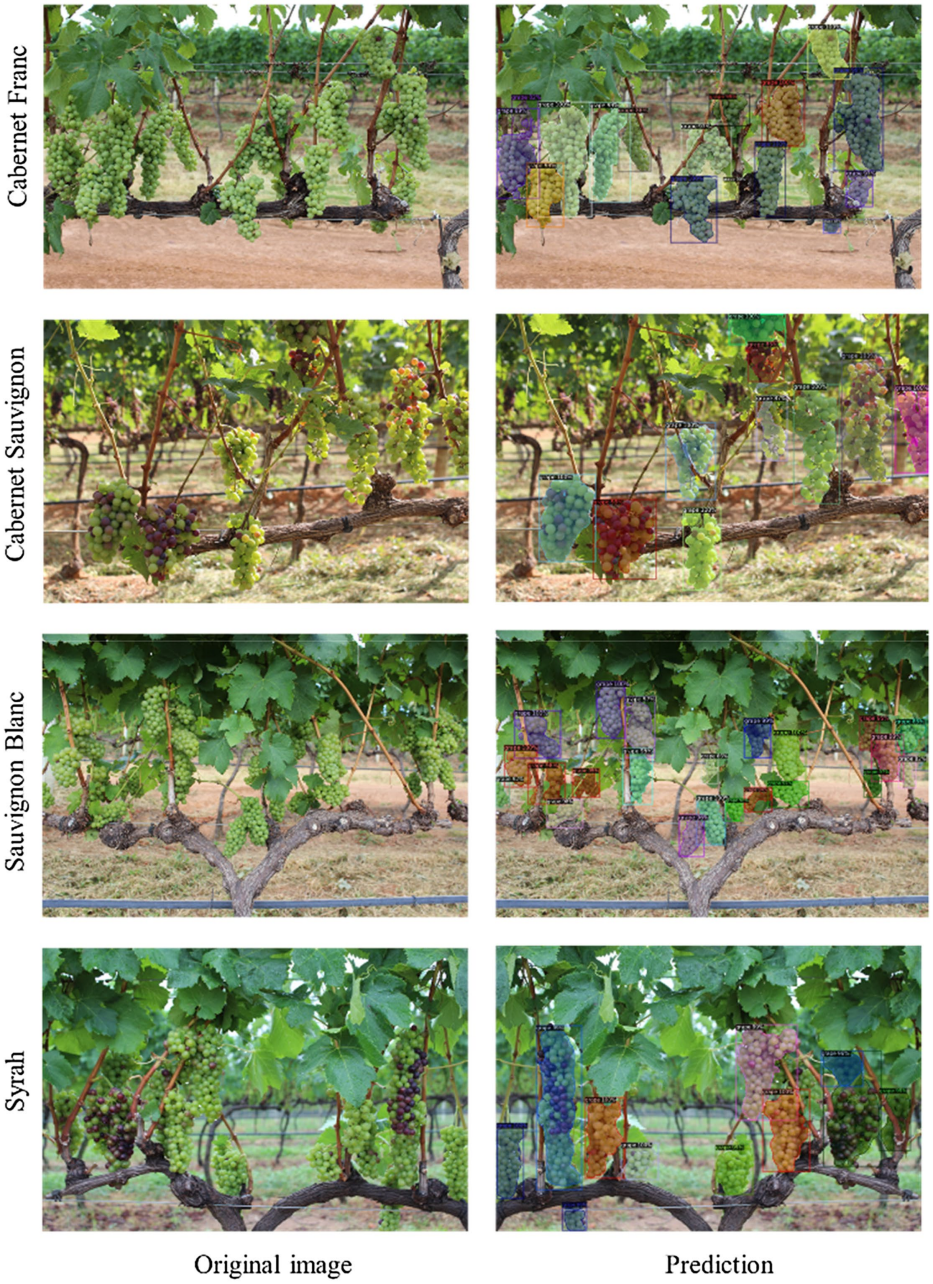
Example results of the improved Mask R-CNN model for different grape varieties.

To verify the effect of different grape varieties on the segmentation of the proposed model, it was tested by using the *Embrapa Wine Grape Instance Segmentation Dataset* (WGISD), a publicly available dataset provided by Santos et al. (Santos et al., 2019). Four different varieties in the dataset, Cabernet Franc, Cabernet Sauvignon, Sauvignon Blanc and Syrah, were selected for prediction and some of the results are shown in Figure 12. What is surprising is that the model is still able to detect the clusters correctly for different varieties. The proposed model potentially improves the segmentation among different varieties. It also shows that the model has a satisfactory generalization performance for different grape varieties, which reduces the variation between varieties. This is probably benefited from the data enhancement during training, where potentially similar features between varieties are captured, reflecting the real field conditions.

## Conclusions and future work

This paper presents an improved instance segmentation model for grapes in a natural field environment. A new backbone network, ResNet50-FPN-ED, was proposed to improve the Mask R-CNN model by introducing an ECA module in the backbone network and using DUC instead of the traditional nearest neighbor interpolation upsampling method in pyramid feature fusion. By correcting the feature information and recovering more image details in the feature fusion stage, the proposed method is able to improve the missed and false detection caused by the variability of grape cluster shape, illumination and occlusion conditions.

For the object detection task of the improved model, AP reached 60.1%, $AP_{IoU=0.5}$ reached 85.6%, $AP_{IoU=0.75}$ reached 65.1%, and $AR_{\max=100}$ reached 69.5%, which was an improvement of 1.4%, 0.5%, 1.1%, and 2.1%, respectively, over the original Mask R-CNN model. For the instance segmentation task of the improved model, AP reached 59.5%, $AP_{IoU=0.5}$ reached 87.1%, $AP_{IoU=0.75}$ reached 66.9%, and $AR_{\max=100}$ reached 66.9%, which was an improvement of 1.6%, 0.8%, 2.6%, and 1.8%, respectively, over the original model. More instances were recovered in the proposed model than the original one, which improves the detection performance for occlusion and darker areas. To verify the effectiveness of the improved Mask R-CNN model, it was also compared against the instance segmentation network SOLOv2 and the object detection network Faster R-CNN (ResNet50-FPN) under the same test conditions, and the improved Mask R-CNN had a higher AP over Faster R-CNN in object detection task (1.8% higher AP, 1.4% higher $AP_{IoU=0.75},$ and 2.6% higher $AR_{\max=100)}.$ For instance segmentation task, the proposed method has 2.2% higher AP and 2.9% higher $AP_{IoU=0.75}$ than SOLOv2, and it is worth mentioning that $AP_{IoU=0.5}$ is 3.5% higher and $AR_{\max=100}$ is 2.5% higher. Furthermore, the proposed model was trained and tested independently on two different datasets, Grape-A and Grape-B. The accuracy of both models was improved to a certain degree compared to the original Mask R-CNN model, and the effect of different datasets on model performance was discussed, and better generalization performance was also achieved across grape varieties on the public dataset.

There is also room for further improvement. One urgent improvement is the model detection speed so that the model can be deployed on mobile robots or agricultural tractor platforms for real time applications with video input. It is noted that the computation cost of the developed model is not a critical issue for offline applications such as yield prediction or yield mapping. However, for other applications in vineyard precision cultivation, such as precision spraying and harvesting, real-time processing is generally required. In the future, the model complexity can be reduced by pruning the model channels, thus increasing the model detection speed. In addition, the evolution of computing hardware and the development of efficient algorithms could also overcome this issue in the future. Furthermore, the manual annotation dataset is limited. Extending the grape dataset under different conditions, using domain adaptation algorithms to improve the generality of the algorithm and investigating further improvements in segmentation accuracy are also necessary for future work.

## Data availability statement

The raw data supporting the conclusions of this article will be made available by the authors, without undue reservation.

## Author contributions

LS and BS: conceptualization. LS: methodology, formal analysis, and writing—original draft preparation. LS, RH, and WQ: investigation. LS, JS, RH, WQ, BS, YS, and YF: writing—review and editing. YS and YF: supervision. BS: project administration and funding acquisition. All authors contributed to the article and approved the submitted version.

## Funding

This work was supported by the National Key R&D Program Project of China (grant no. 2019YFD1002500), Guangxi Key R&D Program Project (grant no. Gui Ke AB21076001), and the Shaanxi Provincial Key R&D Program Project (grant no. 2021NY-041).

## Conflict of interest

The authors declare that the research was conducted in the absence of any commercial or financial relationships that could be construed as a potential conflict of interest.

## Publisher’s note

All claims expressed in this article are solely those of the authors and do not necessarily represent those of their affiliated organizations, or those of the publisher, the editors and the reviewers. Any product that may be evaluated in this article, or claim that may be made by its manufacturer, is not guaranteed or endorsed by the publisher.
